# Reducing Social Isolation and Loneliness Through Creative Aging Initiatives

**DOI:** 10.1093/geroni/igaf122.2133

**Published:** 2025-12-31

**Authors:** Candace Cramer, Robyn Stone

## Abstract

This symposium aligns with the Social Research, Policy, and Practice Section’s focus on social, economic, and environmental contexts of aging by examining the role of arts-based interventions in fostering social connectedness among historically marginalized individuals and communities. Convened by Goddard House, a non-profit provider of assisted living and memory care which also offers creative aging programs in underserved neighborhoods throughout Greater Boston, this session will contribute to ongoing discourse on the implementation and evaluation of community-engaged interventions that enhance quality of life for older adults. Candace Cramer, MA, MBA, CEO of Goddard House, will moderate the symposium and provide an overview of Goddard House’s philosophy of creative aging programs. Katherine Richman, PhD, and Caitlin Coyle, PhD, will summarize evaluation findings of MusicWorks, a program offered at affordable senior housing sites in Boston that fosters community through music that resonates with a culturally and economically diverse group of older adults. Eileen Tell, MPH, and Jacob Watson, EdM, will provide a summary of their comprehensive literature review and environmental scan—the first since 2015—highlighting the evidence base on the effectiveness of creative engagement strategies in reducing social isolation and loneliness. This summary, in conjunction with the findings of the MusicWorks evaluation, demonstrates new and promising approaches to reduce social isolation and loneliness among older adults. Robyn Stone, DrPH, will serve as a discussant, providing a critical analysis of how community-based participatory arts programs can inform broader policy and practice to enhance the well-being of older adults who face systemic barriers to social inclusion.

